# The CALLY index as a novel predictor of left ventricular aneurysm in patients with ST-segment elevation myocardial infarction

**DOI:** 10.3389/fcvm.2026.1882959

**Published:** 2026-07-28

**Authors:** Qinshuo Zhao, Mengqing Li, Dong Hu, Dong Wang, Xin Wang, Yi Li

**Affiliations:** 1 Department of Cardiology, Renmin Hospital of Wuhan University, Wuhan, China; 2 Department of General Medicine, Wuhan University Taikang Tongji Clinical College, Taikang Tongji (Wuhan) Hospital, Wuhan, China; 3 Department of Cardiology, the Central Hospital of Wuhan, Tongji Medical College, Huazhong University of Science and Technology, Wuhan, China; 4 Department of Cardiology, Wuhan No. 1 Hospital, Wuhan Hospital of Traditional Chinese and Western Medicine, Wuhan, China; 5 Department of Cardiometabolic Medicine, Renmin Hospital of Wuhan University, Wuhan, China; 6 Department of Hematology, Renmin Hospital of Wuhan University, Wuhan, China

**Keywords:** C-reactive protein–albumin–lymphocyte index, inflammatory biomarker, left ventricular aneurysm, nomogram, risk prediction model, ST-segment elevation myocardial infarction

## Abstract

**Background:**

Left ventricular aneurysm (LVA) is a serious mechanical complication following ST-segment elevation myocardial infarction (STEMI). The C-reactive protein–albumin–lymphocyte index (CALLY) is a novel composite inflammatory biomarker, but its predictive value for LVA remains unclear.

**Methods:**

We retrospectively enrolled 1,507 STEMI patients who underwent primary percutaneous coronary intervention (pPCI) between January 2020 and March 2026. Patients were divided into quartiles based on CALLY levels. Restricted cubic spline (RCS) analysis was used to explore the dose–response relationship. Least absolute shrinkage and selection operator (LASSO) regression was used for variable screening. Multivariable logistic regression was performed to identify independent predictors. A nomogram was built and validated using receiver operating characteristic (ROC) curves, bootstrap calibration curves, and decision curve analysis (DCA).

**Results:**

LVA occurred in 157 patients (10.42%). The incidence of LVA declined gradually with increasing CALLY quartiles (27.32%, 7.18%, 3.98%, 3.18%; *P* < 0.001). RCS verified a nonlinear inverse association between CALLY and LVA risk (*P* for nonlinearity<0.05). After adjustment, CALLY (Q4 vs. Q1: OR = 0.32, 95%CI: 0.15–0.69, *P* = 0.003), Killip class ≥2, infarct-related artery-left anterior descending (IRA-LAD), left ventricular ejection fraction (LVEF), and cardiac troponin I (cTnI) ≥ 50 ng/mL were independent predictors of LVA. The nomogram based on these five predictors showed good discrimination (AUC=0.931), acceptable calibration, and favorable clinical net benefit.

**Conclusion:**

A lower admission CALLY index was independently associated with a higher risk of LVA in STEMI patients treated with pPCI. The CALLY-based nomogram supports reliable risk stratification and may assist with early clinical decision-making.

## Introduction

ST-segment elevation myocardial infarction (STEMI) is a leading cause of cardiovascular morbidity and mortality worldwide (1). Although pPCI improves survival, a considerable number of patients still develop adverse ventricular remodeling and progress to LVA (2). LVA is characterized by persistent outward bulging of the left ventricular wall throughout the cardiac cycle (3). It arises from localized myocardial necrosis and fibrosis after myocardial infarction (4). LVA profoundly disturbs cardiac geometry and function, predisposing patients to refractory heart failure (5), malignant arrhythmias (6), and thromboembolic events (7). Early identification of patients at high risk for LVA is important to guide close monitoring, timely medical therapy, and revascularization strategies.

Currently, the diagnosis of LVA mainly depends on cardiac imaging modalities, including echocardiography and cardiac magnetic resonance (CMR) imaging (8, 9). However, these imaging techniques are limited by delayed availability, high cost, or limited accessibility in the acute setting, which may hinder early risk stratification. Furthermore, these imaging parameters capture established structural damage but provide limited insight into the upstream biological processes—specifically inflammation, nutritional status, and immune function—that drive adverse ventricular remodeling in the acute phase. Simple, inexpensive, and rapidly available biomarkers are therefore needed to enable early risk stratification for LVA.

The pathogenesis of post-infarction ventricular remodeling is increasingly recognized as a triad of sustained inflammation (10), nutritional depletion (11), and immune dysregulation (12, 13). C-reactive protein (CRP), an acute-phase reactant synthesized by the liver, mirrors the intensity of the systemic inflammatory response (14). Serum albumin, conversely, reflects nutritional reserve and endogenous anti-inflammatory capacity (15), and lymphocyte count reflects immune function and tissue repair potential (16). The CALLY index is a novel composite biomarker integrating these three key pathophysiological dimensions, has shown promise in predicting outcomes in various cardiovascular diseases (17, 18). However, its predictive value for LVA in STEMI patients remains unexplored.

The present study aimed to examine the association between the admission CALLY index and the risk of LVA in STEMI patients treated with pPCI. We hypothesized that a lower CALLY index, reflecting increased inflammation, poorer nutritional status, and impaired immunity, would be independently associated with a higher risk of LVA. We further constructed and validated a nomogram integrating CALLY with established clinical predictors to support early individualized risk stratification of LVA.

## Methods

### Study design and population

This retrospective study consecutively enrolled patients diagnosed with STEMI who underwent pPCI at Renmin Hospital of Wuhan University between January 2020 and March 2026. STEMI was diagnosed according to the criteria outlined in the fourth universal definition of myocardial infarction (19).

**Figure 1:**
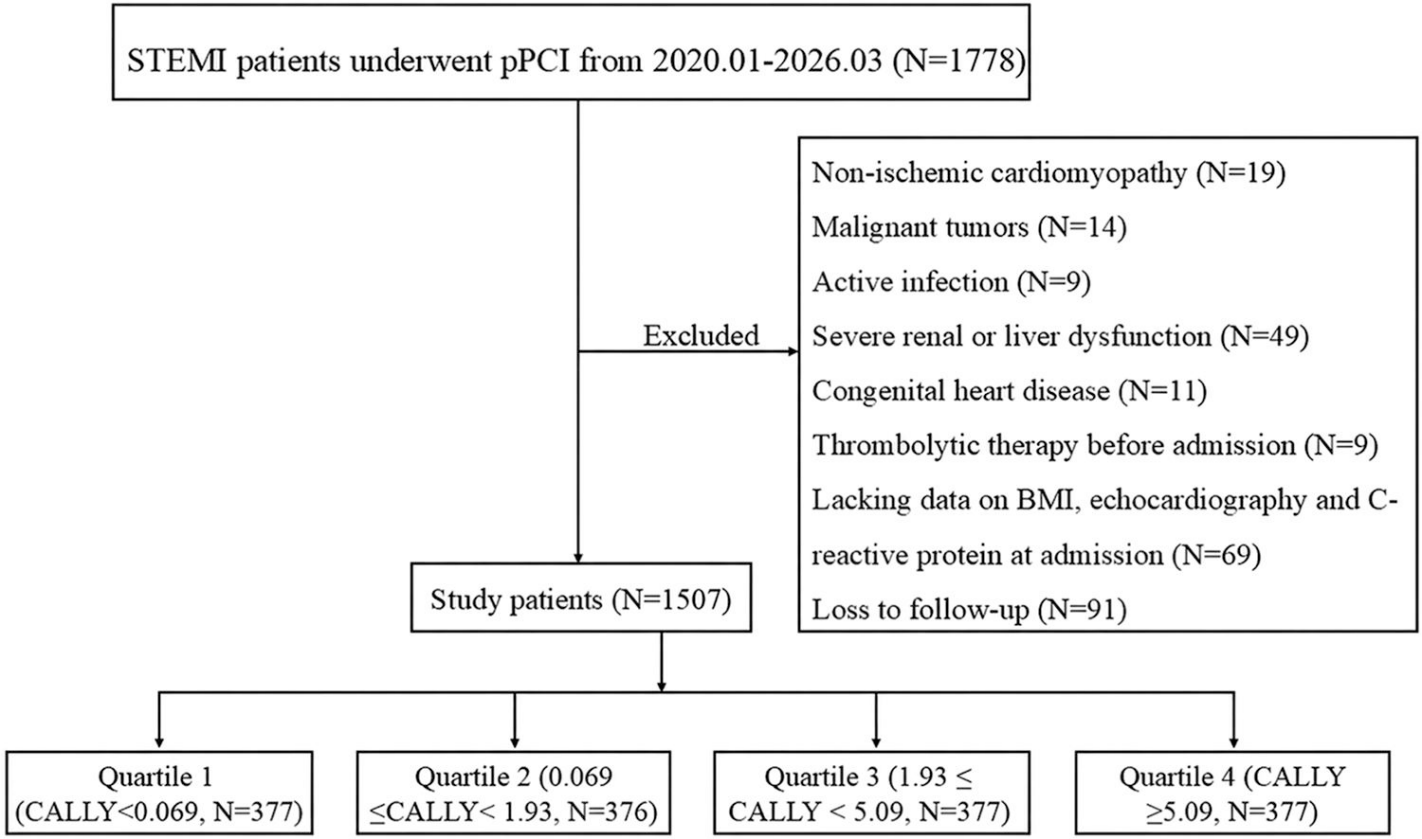
Study flowchart. STEMI, ST-segment elevation myocardial infarction; pPCI, primary percutaneous coronary intervention; CALLY, C-reactive protein-albumin-lymphocyte index; BMI, body mass index.

Of the 1,778 initially screened patients, 271 were excluded for the following reasons: non-ischemic cardiomyopathy (*n* = 19), malignant tumors (*n* = 14), active infection (*n* = 9), severe renal or liver dysfunction (*n* = 49), congenital heart disease (*n* = 11), thrombolytic therapy before admission (*n* = 9), missing data on body mass index (BMI), echocardiography, or admission CRP (*n* = 69), and loss to follow-up (*n* = 91). The remaining 1,507 patients constituted the final cohort. Patients were included in the final cohort only when sufficient clinical, laboratory, and echocardiographic information was available for exposure and endpoint assessment. Patients lacking essential data required for analysis were excluded separately from those lost to follow-up. According to the distribution of the CALLY index, patients were divided into four quartiles: Q1 (CALLY < 0.069, *n* = 377), Q2 (0.069 ≤ CALLY < 1.93, *n* = 376), Q3 (1.93 ≤ CALLY < 5.09, *n* = 377), and Q4 (CALLY ≥ 5.09, *n* = 377). The detailed patient selection flowchart is presented in Figure 1.

### Data collection and definitions

Demographic characteristics (age, sex, BMI), vital signs [heart rate (HR), systolic and diastolic blood pressure], and medical history—including hypertension (HBP), diabetes mellitus (DM), hyperlipidemia, coronary heart disease (CHD), prior coronary stent implantation, drinking, and smoking—were extracted from electronic medical records.

Laboratory data collected within 24 h of admission included white blood cell (WBC) count, neutrophil and lymphocyte counts, monocytes, platelets, red blood cells (RBC), hemoglobin, albumin, alanine aminotransferase (ALT), aspartate aminotransferase (AST), uric acid (UA), creatinine, urea, estimated glomerular filtration rate (eGFR), fasting blood glucose (FBG), triglycerides (TG), total cholesterol (TC), low-density lipoprotein cholesterol (LDL-C), high-density lipoprotein cholesterol (HDL-C), cardiac troponin I (cTnI), NT-proBNP, CRP, creatine kinase (CK), lactate dehydrogenase (LDH), glycated hemoglobin (HbA1c), fibrinogen (FIB), and D-dimer.

Coronary angiographic variables comprised the IRA-LAD, multivessel disease (MVD), Killip class, and Gensini score. Clinical management variables included symptom-to-balloon (S2B) time, use of cardiac assist devices, and stent implantation. Discharge medications were also recorded.

The CALLY index was calculated as: CALLY index = [albumin (g/L) × lymphocyte count (×10⁹/L)]/[CRP (mg/L) × 10]. The primary endpoint was LVA detected during the index hospitalization or scheduled post-discharge echocardiographic follow-up. Transthoracic echocardiography was performed within 72 h after pPCI and repeated during follow-up, mainly at approximately 1 month after discharge; 3-month follow-up echocardiographic data were available in a subset of patients. The median follow-up duration was 1.5 months.

### Echocardiographic assessments

Transthoracic echocardiography was performed by experienced cardiologists within 72 h after pPCI, and repeated at 1 and 3 months during follow-up. LVEF was determined using the biplane Simpson's method. LVEDD and regional wall motion were assessed. LVA was defined as a true left ventricular aneurysm characterized by localized thinning and akinetic or dyskinetic outward bulging of the infarcted myocardial wall. Left ventricular pseudoaneurysm was not considered an endpoint in this study. All diagnoses of LVA were confirmed by two independent cardiologists who were blinded to the patients’laboratory data and CALLY index.

A small number of patients underwent CMR imaging or left ventriculography during subsequent follow-up for clinical indications. However, these examinations were not systematically performed across the entire cohort and were therefore not used as the primary endpoint adjudication method. To ensure consistency of outcome assessment, LVA was adjudicated primarily based on standardized transthoracic echocardiography.

### Coronary angiography and clinical management

Coronary angiography was performed to identify the infarct-related artery (IRA), which was categorized as the left anterior descending artery (LAD), left circumflex artery (LCX), or right coronary artery (RCA). The severity of coronary lesions was quantified using the Gensini score (20). MVD was defined as stenosis of ≥50% in at least two major epicardial vessels. Procedural details of pPCI have been reported previously (21). The S2B time was recorded as the interval from chest pain onset to first balloon inflation or stent deployment. Hemodynamic status on admission was graded using the Killip classification.

Standard periprocedural antithrombotic therapy included aspirin, a P2Y12 inhibitor (clopidogrel or ticagrelor), and intravenous unfractionated heparin. Mechanical circulatory support, including intra-aortic balloon pump (IABP) and extracorporeal membrane oxygenation (ECMO), was implemented in patients with cardiogenic shock or refractory hemodynamic instability, at the discretion of the interventional cardiologist.

Discharge medications were recorded from electronic medical records. Standard post-STEMI medical therapy included dual antiplatelet therapy, statins, *β*-blockers, angiotensin-converting enzyme inhibitors, angiotensin receptor blockers or angiotensin receptor-neprilysin inhibitors, and mineralocorticoid receptor antagonists, as clinically indicated. The use of these therapies was determined by the treating physicians according to guideline recommendations, hemodynamic status, left ventricular function, renal function, serum potassium level, and contraindications. Because these medications were generally prescribed during hospitalization or at discharge and were influenced by clinical severity, they were not included as candidate predictors in the admission-based nomogram model.

### Nomogram construction and validation

We developed a nomogram from the multivariate logistic regression model using the rms package in R. The final model retained independent predictors, each assigned a weighted point score; the total points mapped to the predicted probability of LVA.

Discrimination was assessed using the area under the receiver operating characteristic curve (AUC). Calibration was assessed using calibration curves with 1,000 bootstrap resamples to evaluate the consistency between predicted and observed probabilities of LVA.

DCA was performed to quantify the net benefit of applying the nomogram across a range of threshold probabilities, comparing the model against treat-all and treat-none strategies.

### Statistical analysis

Continuous variables were expressed as mean ± standard deviation or median with interquartile range according to data distribution, and compared using Student's t-test or Mann–Whitney U test as appropriate. Categorical variables were presented as numbers and percentages, and compared using the chi-square test.

Patients with missing essential data required for exposure calculation or endpoint adjudication, including admission CRP or echocardiographic information, were excluded from the final cohort. For other covariates with a low proportion of missing values, multiple imputation was used to reduce potential bias.

RCS analysis was applied to explore the nonlinear dose–response relationship between continuous CALLY levels and LVA risk. Univariate and multivariate binary logistic regression analyses were performed to identify independent predictors of LVA. Variables with *P* < 0.10 in univariate analysis or clinical relevance were entered into the multivariate model. Results were reported as odds ratios (OR) with 95% confidence intervals (CI).

All statistical analyses were performed using SPSS 26.0 and R software (version 4.2.1). A two-sided *P* < 0.05 was considered statistically significant.

## Results

### Study population and baseline characteristics

 Table 1 summarizes baseline characteristics according to quartiles of the correctly calculated admission CALLY index. Patients in Q1 were older (61.65 ± 12.60 years), had lower BMI, and higher HR compared with those in Q4 (all *P* < 0.05). A clear gradient was observed for laboratory markers of inflammation and nutritional status: Q1 patients had the highest levels of WBC, neutrophils, CRP, fibrinogen, and D-dimer, alongside the lowest lymphocyte counts, albumin, hemoglobin, and RBC counts (all *P* < 0.001). Echocardiographic parameters also differed markedly; Q1 patients displayed the lowest LVEF (LVEF: 47.50 ± 8.35%) and the largest LVEDD (LVEDD: 48.23 ± 5.56 mm). Furthermore, Q1 patients had the highest prevalence of Killip class ≥ 2 (41.38%), IRA-LAD (58.09%), and multivessel disease (19.10%), as well as the longest S2B time. Notably, the incidence of LVA decreased steeply across quartiles: 27.32% in Q1, 7.18% in Q2, 3.98% in Q3, and 3.18% in Q4 (*P* < 0.001).

**Table 1:** Baseline characteristics of the study population stratified by quartiles of the admission CALLY index.

Variables	Total (*n* = 1,507)	Q1 (*n* = 377)	Q2 (*n* = 376)	Q3 (*n* = 377)	Q4 (*n* = 377)	Statistic	*P-*value
Demographics							
Age (years)	58.74 ± 12.52	61.65 ± 12.60	59.40 ± 12.75	57.10 ± 11.85	56.82 ± 12.31	F = 12.50	<0.001
Gender (male, %)	1,278 (84.80)	304 (80.64)	316 (84.04)	327 (86.74)	331 (87.80)	*χ*^2^ = 8.97	0.030
BMI (Kg/m2)	24.86 (22.99, 27.04)	24.22 (22.15, 26.45)	25.05 (23.03, 27.06)	25.39 (23.51, 27.36)	24.84 (23.22, 27.04)	χ^2^ = 29.43	<0.001
HR (times/min)	80.00 (72.00, 91.00)	83.00 (74.00, 96.00)	81.00 (72.00, 90.25)	80.00 (72.00, 90.00)	78.00 (69.00, 89.00)	χ^2^ = 24.22	<0.001
SBP (mmHg)	124.03 ± 47.66	120.30 ± 20.99	122.95 ± 19.95	124.82 ± 20.10	128.03 ± 88.45	F = 1.76	0.154
DBP (mmHg)	77.42 ± 13.29	76.07 ± 13.03	78.27 ± 13.76	77.96 ± 13.51	77.37 ± 12.78	F = 2.02	0.109
Medical history, *n* (%)							
HBP	795 (52.75)	208 (55.17)	197 (52.39)	203 (53.85)	187 (49.60)	χ^2^ = 2.59	0.460
DM	321 (21.30)	97 (25.73)	81 (21.54)	73 (19.36)	70 (18.57)	χ^2^ = 6.95	0.074
Hyperlipidemia	130 (8.63)	39 (10.34)	34 (9.04)	32 (8.49)	25 (6.63)	χ^2^ = 3.41	0.333
CHD	165 (10.95)	40 (10.61)	44 (11.70)	44 (11.67)	37 (9.81)	χ^2^ = 0.96	0.810
Coronary Stent Implantation	82 (5.44)	21 (5.57)	25 (6.65)	20 (5.31)	16 (4.24)	χ^2^ = 2.14	0.543
Drinker	876 (58.13)	207 (54.91)	215 (57.18)	234 (62.07)	220 (58.36)	χ^2^ = 4.16	0.245
Smoker	1,077 (71.47)	253 (67.11)	263 (69.95)	283 (75.07)	278 (73.74)	χ^2^ = 7.29	0.063
Laboratory data							
WBC (×10^9^/L)	10.91 (8.96, 13.14)	11.77 (9.54, 13.88)	10.82 (9.16, 12.82)	10.65 (8.78, 13.18)	10.67 (8.71, 12.78)	χ^2^ = 19.72	<0.001
Neutrophil (×10^9^/L)	8.77 (7.03, 11.09)	9.85 (7.73, 11.97)	8.68 (7.42, 10.80)	8.49 (6.70, 11.05)	8.39 (6.52, 10.56)	χ^2^ = 43.47	<0.001
Lymphocyte (×10^9^/L)	1.18 (0.84, 1.63)	0.94 (0.70, 1.35)	1.13 (0.85, 1.53)	1.34 (0.93, 1.89)	1.39 (0.95, 1.80)	χ^2^ = 120.65	<0.001
Monocyte (×10^9^/L)	0.56 (0.39, 0.77)	0.65 (0.42, 0.95)	0.53 (0.39, 0.72)	0.54 (0.38, 0.74)	0.54 (0.37, 0.72)	χ^2^ = 30.37	<0.001
Platelet count (×10^9^/L)	222.00 (183.00, 266.00)	219.00 (179.00, 269.00)	223.00 (187.00, 264.00)	228.00 (185.00, 274.00)	220.00 (185.00, 261.00)	χ^2^ = 4.70	0.195
RBC (×10^9^/L)	4.62 ± 0.60	4.48 ± 0.64	4.63 ± 0.61	4.69 ± 0.57	4.66 ± 0.56	F = 9.42	<0.001
Hemoglobin (g/L)	141.27 ± 18.11	137.12 ± 19.46	141.68 ± 18.44	143.17 ± 16.82	143.11 ± 16.97	F = 9.51	<0.001
Albumin (g/L)	40.25 ± 3.51	38.85 ± 3.74	40.50 ± 3.39	40.84 ± 3.31	40.79 ± 3.22	F = 28.61	<0.001
ALT (U/L）	38.22 (25.00, 60.00)	44.00 (26.00, 68.00)	38.23 (25.00, 59.62)	38.00 (24.00, 58.60)	35.00 (21.83, 51.00)	χ^2^ = 23.46	<0.001
AST (U/L）	123.00 (53.00, 247.50)	169.00 (69.00, 326.00)	131.02 (60.75, 249.00)	116.71 (49.00, 236.00)	88.00 (42.00, 192.00)	χ^2^ = 45.22	<0.001
UA (umol/L)	372.00 (315.00, 441.04)	383.00 (330.00, 459.00)	371.00 (311.50, 441.25)	368.00 (315.00, 434.00)	363.00 (310.00, 430.00)	χ^2^ = 10.33	0.016
Cr (umol/L)	71.00 (61.00, 83.97)	76.00 (63.00, 95.00)	72.00 (61.00, 87.00)	68.92 (60.00, 79.00)	69.00 (59.00, 80.00)	χ^2^ = 43.50	<0.001
Urea (mmol/L)	5.79 (4.70, 7.00)	6.21 (5.06, 7.81)	5.90 (4.89, 7.11)	5.50 (4.61, 6.64)	5.63 (4.50, 6.52)	χ^2^ = 46.59	<0.001
eGFR (mL/min/1.73 m^2^)	96.20 (83.07, 106.25)	90.52 (70.33, 101.77)	95.44 (80.00, 106.17)	99.56 (89.76, 108.12)	98.65 (88.01, 108.54)	χ^2^ = 76.82	<0.001
FBG (mmol/L)	7.09 (6.01, 9.19)	7.58 (6.30, 10.44)	7.15 (6.00, 9.66)	7.03 (6.04, 8.96)	6.74 (5.79, 8.05)	χ^2^ = 34.58	<0.001
TG (mmol/L)	0.95 (0.62, 1.58)	0.94 (0.64, 1.41)	1.00 (0.63, 1.64)	0.93 (0.62, 1.67)	0.92 (0.60, 1.69)	χ^2^ = 2.90	0.407
TC (mmol/L)	4.54 (3.89, 5.22)	4.58 (3.80, 5.35)	4.62 (3.98, 5.24)	4.58 (3.96, 5.24)	4.40 (3.79, 5.09)	χ^2^ = 8.52	0.036
LDL-C (mmol/L)	2.86 (2.27, 3.47)	2.88 (2.31, 3.54)	2.88 (2.35, 3.47)	2.96 (2.32, 3.56)	2.82 (2.14, 3.35)	χ^2^ = 9.54	0.023
HDL-C (mmol/L)	0.98 (0.83, 1.16)	0.96 (0.82, 1.16)	0.99 (0.86, 1.15)	0.99 (0.83, 1.14)	0.99 (0.84, 1.17)	χ^2^ = 0.90	0.825
cTnI>50 (ng/mL)	827 (54.88)	251 (66.58)	209 (55.59)	190 (50.40)	177 (46.95)	χ^2^ = 33.54	<0.001
NT-proBNP (pg/mL)	245.50 (82.50, 954.00)	1,016.00 (247.00, 3,172.00)	233.50 (78.46, 804.50)	170.00 (74.00, 553.00)	127.00 (49.00, 364.00)	χ^2^ = 231.34	<0.001
CRP (mg/L)	2.75 (0.98, 6.49)	12.53 (7.64, 26.25)	3.82 (2.81, 5.43)	1.78 (1.11, 2.65)	0.53 (0.32, 0.84)	χ^2^ = 1,217.17	<0.001
CK (U/L)	1,289.00 (569.50, 2,904.00)	1,571.00 (674.00, 3,510.71)	1,433.27 (739.25, 3,007.35)	1,266.00 (548.00, 2,788.00)	1,023.00 (402.30, 2,248.00)	χ^2^ = 29.43	<0.001
LDH (U/L)	398.20 (257.50, 635.22)	564.00 (340.00, 842.00)	390.00 (276.00, 632.00)	387.00 (243.00, 564.00)	310.40 (232.00, 509.93)	χ^2^ = 112.03	<0.001
HbAlc (%)	6.55 ± 1.43	6.74 ± 1.57	6.61 ± 1.50	6.57 ± 1.42	6.26 ± 1.17	F = 7.67	<0.001
FIB (g/L)	2.85 (2.42, 3.38)	3.43 (2.84, 4.47)	2.89 (2.47, 3.36)	2.76 (2.40, 3.15)	2.55 (2.24, 2.95)	χ^2^ = 219.51	<0.001
D-Dimer (mg/L)	0.36 (0.21, 0.67)	0.54 (0.29, 1.08)	0.40 (0.23, 0.71)	0.31 (0.20, 0.51)	0.29 (0.19, 0.48)	χ^2^ = 108.14	<0.001
Echocardiography							
AAOD (mm)	33.53 ± 3.52	33.85 ± 3.55	33.63 ± 3.56	33.44 ± 3.37	33.21 ± 3.56	F = 2.27	0.079
LAD (mm)	36.00 (33.00, 38.00)	36.00 (33.00, 39.00)	36.00 (33.00, 38.00)	35.00 (33.00, 38.00)	35.00 (32.00, 38.00)	χ^2^ = 18.53	<0.001
LVEDD (mm)	47.02 ± 4.52	48.23 ± 5.56	46.80 ± 4.29	46.71 ± 4.17	46.33 ± 3.60	F = 13.02	<0.001
RAD (mm)	34.00 (32.00, 36.00)	34.00 (31.00, 36.00)	34.00 (32.00, 36.00)	33.00 (31.00, 36.00)	34.00 (32.00, 36.00)	χ^2^ = 3.34	0.342
IVSD (mm)	10.24 ± 1.23	10.17 ± 1.20	10.32 ± 1.37	10.24 ± 1.18	10.22 ± 1.15	F = 1.00	0.393
LVEF (%)	51.03 ± 7.53	47.50 ± 8.35	51.36 ± 7.13	51.82 ± 6.79	53.43 ± 6.47	F = 45.61	<0.001
Medication at hospital discharge, *n* (%)							
Aspirin/Indobufen	1,448 (96.08)	355 (94.16)	364 (96.81)	363 (96.29)	366 (97.08)	χ^2^ = 5.26	0.154
Clopidogrel	427 (28.33)	144 (38.20)	109 (28.99)	74 (19.63)	100 (26.53)	χ^2^ = 32.81	<0.001
Ticagrelor	1,071 (71.07)	231 (61.27)	265 (70.48)	299 (79.31)	276 (73.21)	χ^2^ = 30.95	<0.001
Statins	1,503 (99.73)	373 (98.94)	376 (100.00)	377 (100.00)	377 (100.00)	-	0.015
*β*-blockers	1,179 (78.23)	303 (80.37)	294 (78.19)	304 (80.64)	278 (73.74)	χ^2^ = 6.76	0.080
Thiazide/loop diuretic	442 (29.33)	181 (48.01)	109 (28.99)	85 (22.55)	67 (17.77)	χ^2^ = 96.16	<0.001
Spironolactone	518 (34.37)	198 (52.52)	129 (34.31)	100 (26.53)	91 (24.14)	χ^2^ = 82.84	<0.001
ACEI	129 (8.56)	35 (9.28)	30 (7.98)	27 (7.16)	37 (9.81)	χ^2^ = 2.11	0.549
ARB	800 (53.09)	196 (51.99)	189 (50.27)	216 (57.29)	199 (52.79)	χ^2^ = 4.08	0.253
Insulin	103 (6.83)	28 (7.43)	38 (10.11)	20 (5.31)	17 (4.51)	χ^2^ = 11.12	0.011
OADS	393 (26.08)	115 (30.50)	109 (28.99)	90 (23.87)	79 (20.95)	χ^2^ = 11.57	0.009
Coronary artery injury							
IRA-LAD, *n* (%)	783 (51.96)	219 (58.09)	203 (53.99)	194 (51.46)	167 (44.30)	χ^2^ = 15.20	0.002
MVD, (%)	198 (13.14)	72 (19.10)	53 (14.10)	44 (11.67)	29 (7.69)	χ^2^ = 22.54	<0.001
Killip class ≥2, *n* (%)	338 (22.43)	156 (41.38)	71 (18.88)	60 (15.92)	51 (13.53)	χ^2^ = 106.90	<0.001
Gensini score	50.00 (35.00, 80.00)	58.00 (41.00, 84.00)	51.00 (35.75, 80.00)	50.00 (36.00, 80.00)	43.00 (30.00, 58.00)	χ^2^ = 61.44	<0.001
LVA, *n* (%)	157 (10.42)	103 (27.32)	27 (7.18)	15 (3.98)	12 (3.18)	χ^2^ = 157.53	<0.001
LVT, *n* (%)	68 (4.51)	33 (8.75)	17 (4.52)	11 (2.92)	7 (1.86)	χ^2^ = 24.13	<0.001
Clinical management							
S2B (h)	5.00 (2.00, 10.00)	7.00 (3.50, 15.00)	4.00 (2.00, 10.00)	4.00 (2.00, 8.00)	4.00 (2.00, 7.00)	χ^2^ = 62.48	<0.001
Cardiac assist device, *n* (%)	209 (13.87)	97 (25.73)	49 (13.03)	33 (8.75)	30 (7.96)	χ^2^ = 63.91	<0.001
Stent implantation	927 (61.55)	216 (57.29)	246 (65.43)	234 (62.23)	231 (61.27)	χ^2^ = 5.36	0.147

Data are presented as mean ± standard deviation (for normally distributed continuous variables), median [interquartile range] (for non-normally distributed continuous variables), or number (percentage) (for categorical variables). Comparisons across CALLY quartiles were performed using one-way analysis of variance (for normally distributed continuous variables), Kruskal–Wallis H test (for non-normally distributed continuous variables), or chi-square test (for categorical variables). A *P* value < 0.05 was considered statistically significant. The CALLY index quartile ranges were defined as follows: Q1 (CALLY < 0.069), Q2 (0.069 ≤ CALLY < 1.93), Q3 (1.93 ≤ CALLY < 5.09), and Q4 (CALLY ≥ 5.09). CALLY index = [albumin (g/L) × lymphocyte count (×10⁹/L)]/[CRP (mg/L) × 10]. STEMI, ST-segment elevation myocardial infarction; CALLY, C-reactive protein-albumin-lymphocyte index; Q, quartile; BMI, body mass index; SBP, systolic blood pressure; DBP, diastolic blood pressure; HR, heart rate; HBP, hypertension; DM, diabetes mellitus; MI, myocardial infarction; PCI, percutaneous coronary intervention; CABG, coronary artery bypass grafting; WBC, white blood cell count; Neu#, neutrophil count; Lym#, lymphocyte count; Hb, hemoglobin; PLT, platelet count; FBG, fasting blood glucose; HbA1c, glycated hemoglobin; TC, total cholesterol; TG, triglycerides; LDL-C, low-density lipoprotein cholesterol; HDL-C, high-density lipoprotein cholesterol; CRP, C-reactive protein; cTnI, cardiac troponin I; CK, creatine kinase; LDH, lactate dehydrogenase; NT-proBNP, N-terminal pro-B-type natriuretic peptide; Scr, serum creatinine; BUN, blood urea nitrogen; eGFR, estimated glomerular filtration rate; FIB, fibrinogen; D-dimer, D-dimer; IRA, infarct-related artery; LAD, left anterior descending coronary artery; LCX, left circumflex coronary artery; RCA, right coronary artery;S2B time, symptom-to-balloon time; LVEF, left ventricular ejection fraction; LVEDD, left ventricular end-diastolic diameter; LAD, left atrial diameter; LVT, left ventricular thrombus; LVA, left ventricular aneurysm.

 Table 2 compares baseline characteristics between patients with and without LVA. Among the 1,507 patients, 157 (10.42%) developed LVA. Most LVA cases were first diagnosed during the hospitalization, whereas a small proportion were first detected after discharge during follow-up, mainly at the 1-month and 3-month follow-up. LVA patients were older (62.43 ± 12.69 vs. 58.31 ± 12.44 years, *P* < 0.001), more likely to be female (22.93% vs. 14.30%, *P* = 0.004), and had lower BMI and lower blood pressure. They also presented with more severe inflammatory and metabolic derangements, including higher WBC counts, neutrophils, monocytes, CRP, CK, LDH, fibrinogen, D-dimer, and NT-proBNP, but lower albumin and hemoglobin (all *P* < 0.05). The CALLY index was significantly lower in the LVA group than in the non-LVA group [median 0.39 (IQR 0.17–1.07) vs. 2.18 (0.85–5.55), *P* < 0.001].

**Table 2:** Baseline characteristics of the study population stratified by the presence or absence of LVA.

Variables	Total (*n* = 1,507)	Non-LVA (*n* = 1,350)	LVA (*n* = 157)	Statistic	*P*-value
Demographics					
Age (years)	58.74 ± 12.52	58.31 ± 12.44	62.43 ± 12.69	t = −3.92	<0.001
Gender (male, %)	1,278 (84.80)	1,157 (85.70)	121 (77.07)	χ^2^ = 8.14	0.004
BMI (Kg/m2)	24.86 (22.99, 27.04)	24.97 (23.04, 27.04)	24.22 (21.97, 26.30)	Z = −2.98	0.003
HR (times/min)	80.00 (72.00, 91.00)	79.00 (72.00, 90.00)	90.00 (79.00, 101.00)	Z = −7.20	<0.001
SBP (mmHg)	124.03 ± 47.66	124.99 ± 49.84	115.71 ± 19.18	t = 2.31	0.021
DBP (mmHg)	77.42 ± 13.29	77.82 ± 13.21	73.97 ± 13.49	t = 3.45	<0.001
Medical history, *n* (%)					
HBP	795 (52.75)	718 (53.19)	77 (49.04)	χ^2^ = 0.97	0.325
DM	321 (21.30)	279 (20.67)	42 (26.75)	χ^2^ = 3.11	0.078
Hyperlipidemia	130 (8.63)	117 (8.67)	13 (8.28)	χ^2^ = 0.03	0.870
CHD	165 (10.95)	135 (10.00)	30 (19.11)	χ^2^ = 11.97	<0.001
Coronary Stent Implantation	82 (5.44)	68 (5.04)	14 (8.92)	χ^2^ = 4.12	0.042
Drinker	876 (58.13)	786 (58.22)	90 (57.32)	χ^2^ = 0.05	0.829
Smoker	1,077 (71.47)	971 (71.93)	106 (67.52)	χ^2^ = 1.34	0.247
Laboratory data					
WBC (×109/L)	10.91 (8.96, 13.14)	10.77 (8.88, 12.96)	12.37 (10.46, 14.57)	Z = −5.75	<0.001
Neutrophil (×109/L)	8.77 (7.03, 11.09)	8.65 (6.93, 10.91)	10.17 (8.39, 13.02)	Z = −5.75	<0.001
Lymphocyte (×109/L)	1.18 (0.84, 1.63)	1.19 (0.85, 1.63)	1.11 (0.77, 1.63)	Z = −1.23	0.218
Monocyte (×109/L)	0.56 (0.39, 0.77)	0.54 (0.38, 0.74)	0.74 (0.46, 1.08)	Z = −6.06	<0.001
Platelet count (×109/L)	222.00 (183.00, 266.00)	222.00 (183.25, 265.00)	222.00 (178.00, 272.00)	Z = −0.23	0.816
RBC (×109/L)	4.61 (4.24, 5.01)	4.64 (4.26, 5.02)	4.46 (4.02, 4.91)	Z = −3.65	<0.001
Hemoglobin (g/L)	141.27 ± 18.11	141.98 ± 17.65	135.19 ± 20.72	t = 3.94	<0.001
Albumin (g/L)	40.25 ± 3.51	40.49 ± 3.40	38.16 ± 3.76	t = 8.02	<0.001
CALLY index	1.93 (0.69, 5.09)	2.18 (0.85, 5.55)	0.39 (0.17, 1.07)	Z = −11.42	<0.001
UA (umol/L)	372.00 (315.00, 441.04)	370.00 (314.00, 439.32)	398.91 (326.00, 463.38)	Z = −2.27	0.023
Cr (umol/L)	71.00 (61.00, 83.97)	70.00 (61.00, 83.00)	75.00 (63.00, 99.00)	Z = −3.48	<0.001
Urea (mmol/L)	5.79 (4.70, 7.00)	5.71 (4.67, 6.86)	6.48 (5.35, 8.03)	Z = −5.43	<0.001
eGFR (mL/min/1.73 m^2^)	96.20 (83.07, 106.25)	96.77 (84.81, 106.87)	89.43 (69.85, 101.08)	Z = −5.15	<0.001
FBG (mmol/L)	7.09 (6.01, 9.19)	7.00 (5.98, 9.03)	8.08 (6.54, 11.20)	Z = −4.52	<0.001
TG (mmol/L)	0.95 (0.62, 1.58)	0.94 (0.62, 1.60)	1.02 (0.65, 1.42)	Z = −0.53	0.597
TC (mmol/L)	4.54 (3.89, 5.22)	4.54 (3.90, 5.22)	4.56 (3.72, 5.40)	Z = −0.68	0.499
LDL-C (mmol/L)	2.86 (2.27, 3.47)	2.86 (2.27, 3.46)	2.97 (2.20, 3.61)	Z = −0.53	0.595
HDL-C (mmol/L)	0.98 (0.83, 1.16)	0.98 (0.84, 1.15)	0.99 (0.82, 1.17)	Z = −0.27	0.789
cTnI>50 ng/mL	827 (54.88)	702 (52.00)	125 (79.62)	χ^2^ = 43.32	<0.001
NT-proBNP (pg/mL)	245.50 (82.50, 954.00)	201.50 (73.25, 716.75)	2,414.00 (893.00, 4,824.00)	Z = −12.98	<0.001
CRP(mg/L)	2.75 (0.98, 6.49)	2.44 (0.89, 5.38)	11.19 (4.03, 33.64)	Z = −11.32	<0.001
CK (U/L)	1,289.00 (569.50, 2,904.00)	1,180.50 (543.09, 2,645.38)	2,983.00 (1,172.00, 4,557.00)	Z = −7.05	<0.001
LDH (U/L)	398.20 (257.50, 635.22)	373.00 (248.55, 588.75)	698.00 (474.00, 1,032.00)	Z = −10.87	<0.001
HbAlc (%)	6.55 ± 1.43	6.51 ± 1.42	6.81 ± 1.53	t = −2.49	0.013
FIB (g/L)	2.85 (2.42, 3.38)	2.83 (2.40, 3.30)	3.22 (2.61, 4.30)	Z = −5.48	<0.001
D-Dimer (mg/L)	0.36 (0.21, 0.67)	0.34 (0.21, 0.60)	0.71 (0.36, 1.63)	Z = −8.56	<0.001
Echocardiography					
AAOD (mm)	33.53 ± 3.52	33.47 ± 3.48	34.05 ± 3.75	t = −1.97	0.049
LAD (mm)	36.00 (33.00, 38.00)	35.00 (33.00, 38.00)	37.00 (34.00, 39.00)	Z = −3.25	0.001
LVEDD (mm)	47.02 ± 4.52	46.52 ± 3.95	51.31 ± 6.42	t = −9.13	<0.001
RAD (mm)	34.00 (32.00, 36.00)	34.00 (32.00, 36.00)	34.00 (31.00, 36.00)	Z = −0.30	0.764
IVSD (mm)	10.24 ± 1.23	10.32 ± 1.19	9.60 ± 1.37	t = 6.30	<0.001
LVEF (%)	51.03 ± 7.53	52.35 ± 6.55	39.63 ± 5.53	t = 26.72	<0.001
Medication at hospital discharge, *n* (%)					
Aspirin/Indobufen	1,448 (96.08)	1,307 (96.81)	141 (89.81)	χ^2^ = 18.35	<0.001
Clopidogrel	427 (28.33)	352 (26.07)	75 (47.77)	χ^2^ = 32.60	<0.001
Ticagrelor	1,071 (71.07)	991 (73.41)	80 (50.96)	χ^2^ = 34.48	<0.001
Statins	1,503 (99.73)	1,347 (99.78)	156 (99.36)	-	0.356
β-blockers	1,179 (78.23)	1,052 (77.93)	127 (80.89)	χ^2^ = 0.73	0.394
Thiazide/loop diuretic	442 (29.33)	333 (24.67)	109 (69.43)	χ^2^ = 135.94	<0.001
Spironolactone	518 (34.37)	407 (30.15)	111 (70.70)	χ^2^ = 102.53	<0.001
ACEI	129 (8.56)	114 (8.44)	15 (9.55)	χ^2^ = 0.22	0.638
ARB	800 (53.09)	714 (52.89)	86 (54.78)	χ^2^ = 0.20	0.654
Insulin	103 (6.83)	89 (6.59)	14 (8.92)	χ^2^ = 1.19	0.275
OADS	393 (26.08)	342 (25.33)	51 (32.48)	χ^2^ = 3.73	0.053
Coronary artery injury					
IRA-LAD, *n* (%)	783 (51.96)	644 (47.70)	139 (88.54)	χ^2^ = 93.94	<0.001
MVD, (%)	198 (13.14)	175 (12.96)	23 (14.65)	χ^2^ = 0.35	0.554
Killip class ≥2, *n* (%)	338 (22.43)	224 (16.59)	114 (72.61)	χ^2^ = 253.68	<0.001
Gensini score	50.00 (35.00, 80.00)	48.00 (34.00, 72.00)	82.00 (53.00, 94.00)	Z = −10.10	<0.001
LVT, *n* (%)	68 (4.51)	31 (2.30)	37 (23.57)	χ^2^ = 147.68	<0.001
Clinical management					
S2B (h)	5.00 (2.00, 10.00)	5.00 (2.00, 9.00)	8.00 (4.00, 18.00)	Z = −5.89	<0.001
Cardiac assist device, *n* (%)	209 (13.87)	143 (10.59)	66 (42.04)	χ^2^ = 116.42	<0.001
Stent implantation	927 (61.55)	850 (63.01)	77 (49.04)	χ^2^ = 11.59	<0.001

As for echocardiographic data, LVA patients had lower LVEF (39.63 ± 5.53% vs. 52.35 ± 6.55%) and enlarged LVEDD (51.31 ± 6.42 mm vs. 46.52 ± 3.95 mm, both *P* < 0.001). The LVA group also had a higher burden of coronary disease, evidenced by a higher Gensini score (82 vs. 48), more frequent IRA-LAD involvement (88.54% vs. 47.70%), and higher rates of Killip class ≥ 2 (72.61% vs. 16.59%, all *P* < 0.001).

### Association between the CALLY index and LVA risk

**Figure 2:**
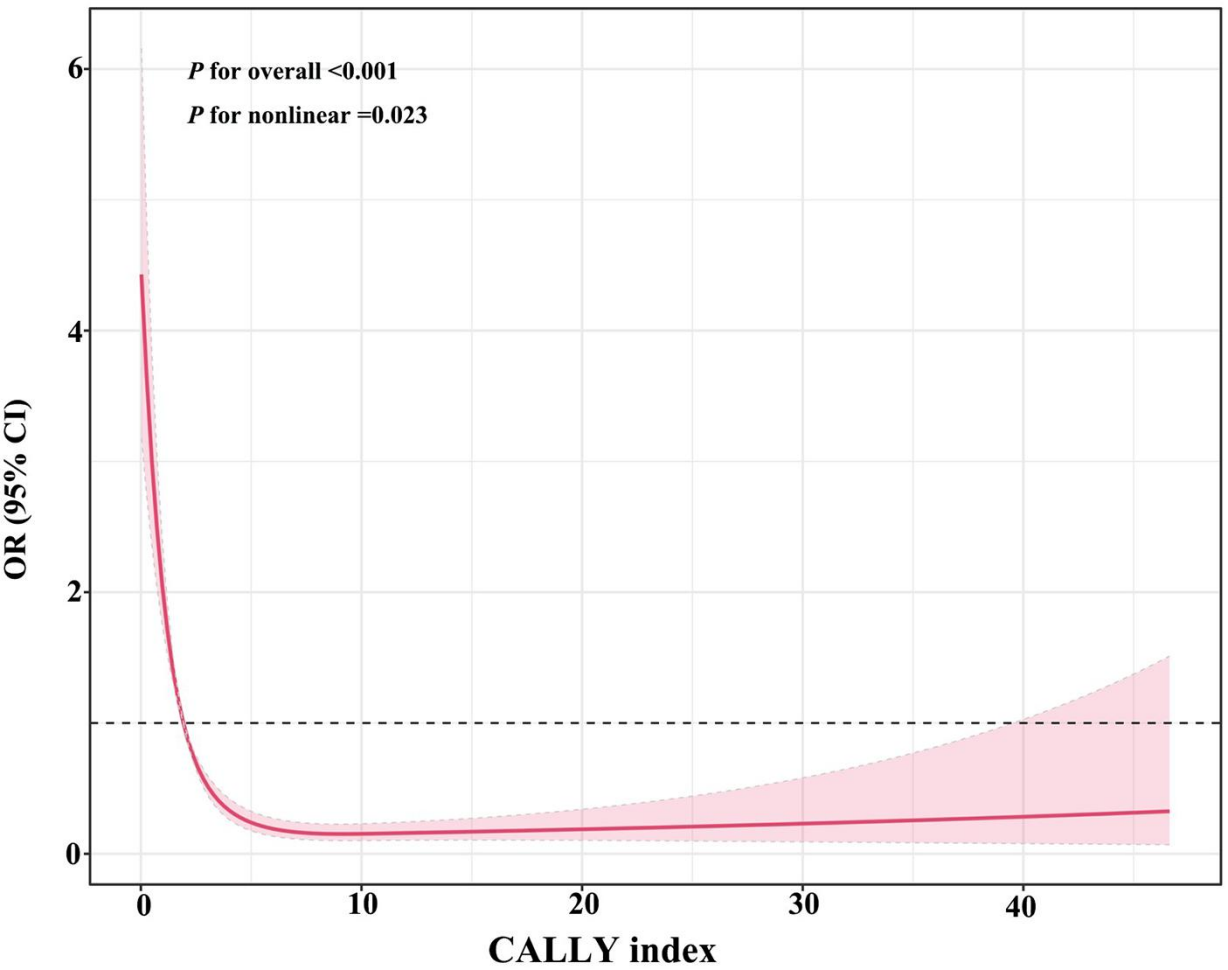
RCS analysis of the association between continuous CALLY index and the risk of LVA. The solid red line represents the adjusted odds ratio (OR), and the shaded pink area indicates the corresponding 95% confidence interval (CI). The horizontal dashed black line denotes the reference level at an OR of 1.0. After multivariate adjustment, there was a significant overall association between the CALLY index and LVA risk (*P* for overall < 0.001), and a statistically significant nonlinear dose–response relationship was also observed (*P* for nonlinea*r* = 0.023). CALLY, C-reactive protein-albumin-lymphocyte index; OR, odds ratio; CI, confidence interval; LVA, left ventricular aneurysm.

RCS analysis revealed a nonlinear, inverse relationship between the continuous CALLY index and LVA risk (*P* for overall association < 0.001; *P* for nonlinearity = 0.023). LVA risk increased significantly when the CALLY index decreased to below 1.0, while the curve plateaued at higher values (Figure 2).

In univariate logistic regression, higher CALLY quartiles were associated with lower LVA risk. Compared with Q1, the unadjusted odds ratios (ORs) were 0.21 (95% CI 0.13–0.32) for Q2, 0.11 (0.06–0.19) for Q3, and 0.09 (0.05–0.16) for Q4 (all *P* < 0.001). After multivariable adjustment, the inverse association remained significant: adjusted ORs were 0.39 (95% CI: 0.21–0.72) for Q2, 0.27 (95% CI: 0.14–0.53) for Q3, and 0.32 (95% CI: 0.15–0.69) for Q4 (all *P* < 0.01) (Table 3).

**Table 3:** Univariate and multivariate logistic regression analyses of predictors for LVA.

Variables	Univariate logistic regression analysis			Multivariate logistic regression analysis		
	OR	95%CI	*P*-value	OR	95%CI	*P*-value
Gender	0.56	0.38∼0.84	0.005			
HBP	0.85	0.61∼1.18	0.326			
Diabetes	1.4	0.96∼2.04	0.079			
cTnI≥50 ng/mL	3.61	2.41∼5.39	<0.001	1.79	1.01∼3.17	0.048
Cardiac assist device	6.12	4.27∼8.78	<0.001			
Killip≥2	13.33	9.12∼19.47	<0.001	2.84	1.73∼4.66	<0.001
IRA-LAD	8.47	5.12∼13.99	<0.001	4.23	2.03∼8.82	<0.001
MVD	1.15	0.72∼1.84	0.554			
CALLY index						
Q1	1	Reference		1	Reference	
Q2	0.21	0.13∼0.32	<0.001	0.39	0.21∼0.72	0.003
Q3	0.11	0.06∼0.19	<0.001	0.27	0.14∼0.53	<0.001
Q4	0.09	0.05∼0.16	<0.001	0.32	0.15∼0.69	0.003
Age (years)	1.03	1.01∼1.04	<0.001			
eGFR (mL/min/1.73 m^2^)	0.98	0.97∼0.99	<0.001	0.99	0.98∼1.00	0.125
LVEDD (mm)	1.22	1.18∼1.27	<0.001	1.05	1.01∼1.10	0.049
LVEF (%)	0.73	0.70∼0.76	<0.001	0.81	0.76∼0.85	<0.001
Gensini score	1.03	1.02∼1.04	<0.001			
S2B(h)	1.05	1.03∼1.06	<0.001			
NT-proBNP (pg/mL)	1.01	1.01∼1.01	<.001	1	1.00∼1.00	0.147

OR, odds ratio; CI, confidence interval; HBP, hypertension; cTnI, cardiac troponin I; Killip, Killip class; IRA-LAD, infarct-related artery as the left anterior descending artery; MVD, multivessel disease; CALLY, CRP–albumin–lymphocyte index; Q1–Q4, quartiles 1 through 4 of the CALLY index; eGFR, estimated glomerular filtration rate; LVEDD, left ventricular end-diastolic dimension; LVEF, left ventricular ejection fraction; S2B, symptom-to-balloon; NT-proBNP, N-terminal pro-B-type natriuretic peptide.

### Model development and final predictors

**Figure 3:**
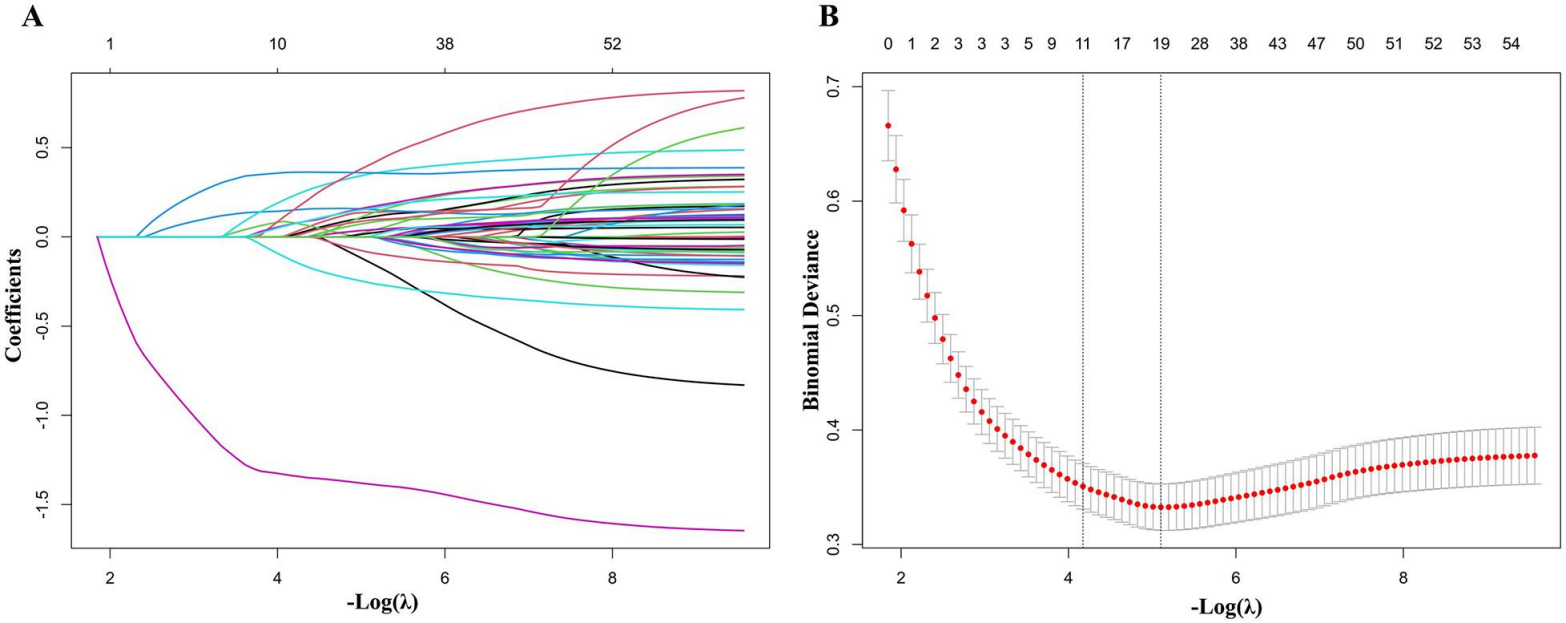
LASSO regression for variable selection. **(A)** LASSO coefficient profiles of all candidate predictive variables. Each colored curve represents the changing coefficient trajectory of an individual variable as the penalty parameter *λ* varies. **(B)** Optimal *λ* value determined via 10-fold cross-validation based on minimum binomial deviance. The two vertical dashed lines indicate the optimal *λ* value selected by the minimum criteria and the 1-standard error criterion, respectively. LASSO, least absolute shrinkage and selection operator; *λ*, tuning penalty parameter.

LASSO regression with 10-fold cross-validation was used for variable selection among candidate predictors. The optimal tuning parameter (*λ*) selected five variables for inclusion in the final multivariate model: the natural logarithm of the CALLY index, Killip class ≥ 2, IRA-LAD, cTnI ≥ 50 ng/mL, and LVEF (Figure 3).

**Figure 4:**
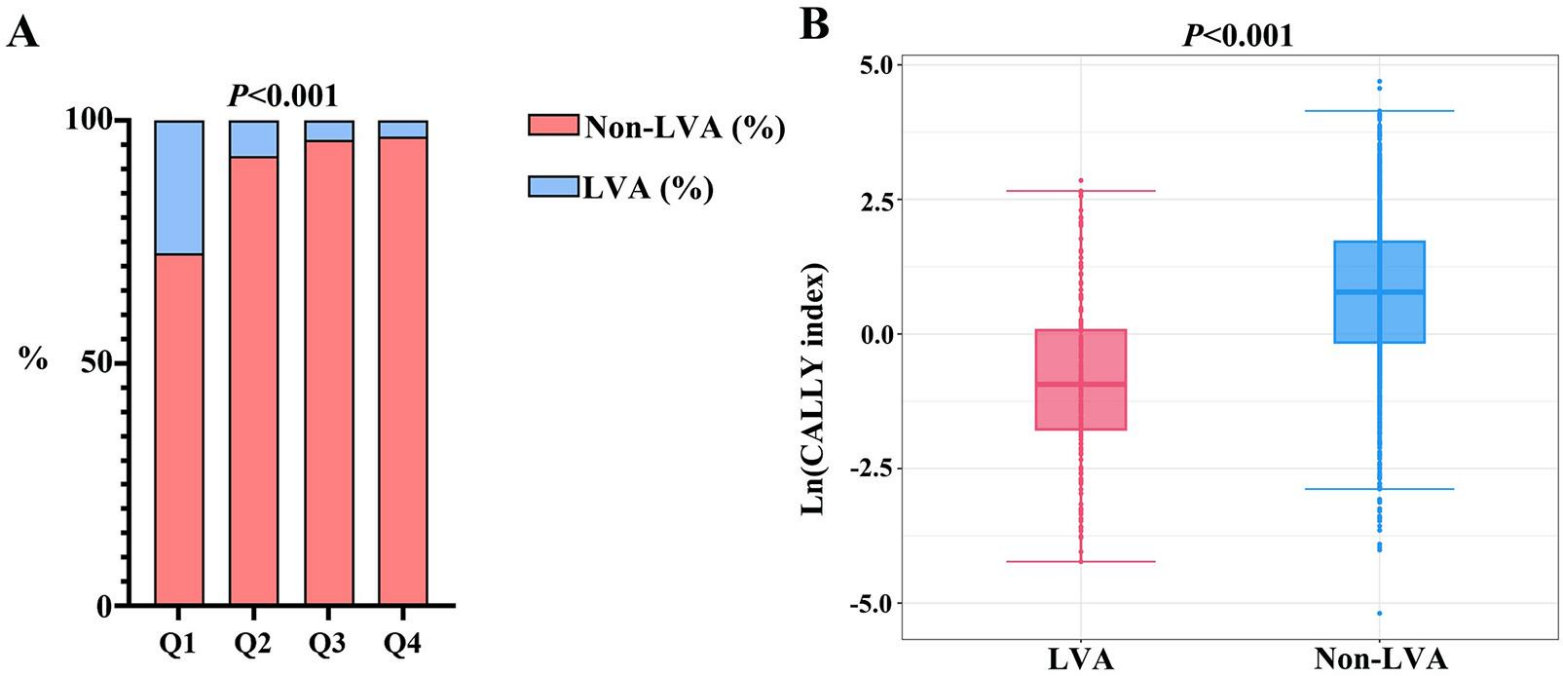
Comparison of LVA prevalence and CALLY index distribution across study groups. **(A)** Stacked bar chart showing the proportion of patients with and without LVA across the four CALLY index quartiles (Q1–Q4). **(B)** Box plot comparing natural logarithm-transformed CALLY index levels between patients with LVA and those without LVA. LVA, left ventricular aneurysm; Non-LVA, patients without left ventricular aneurysm; CALLY, C-reactive protein-albumin-lymphocyte index; Ln, natural logarithm; Q, quartile.

In the final model, lower ln(CALLY), Killip class ≥2, IRA-LAD, cTnI ≥50 ng/mL, and lower LVEF were independently associated with LVA. To maintain consistency between the statistical model and the graphical prediction tool, only these LASSO-selected predictors were included in the final nomogram (Table 3). Consistent with these findings, LVA prevalence decreased across CALLY quartiles, and ln(CALLY) was significantly lower in patients with LVA than in those without LVA (Figure 4).

### Subgroup analyses

**Figure 5:**
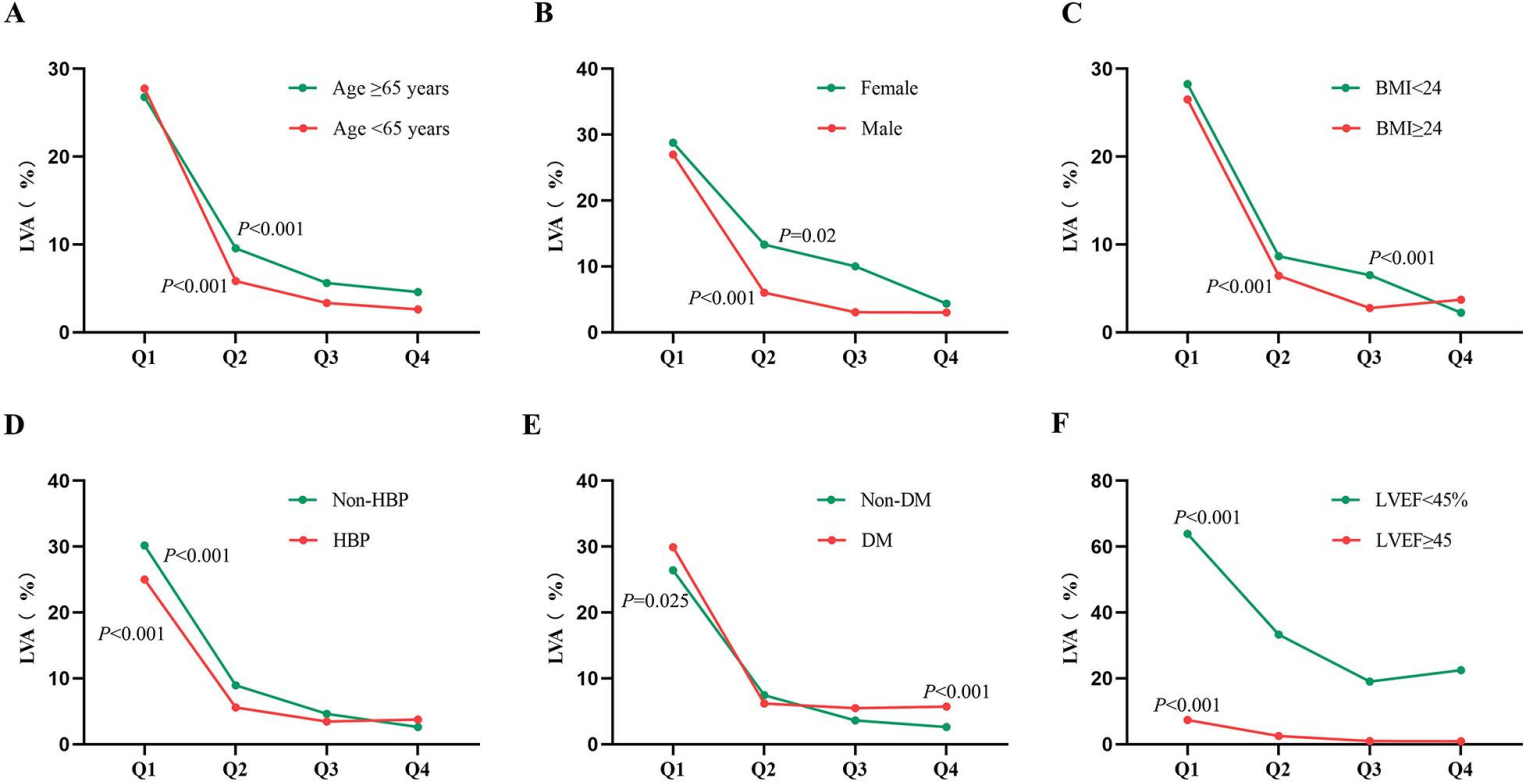
Stratified analysis of the association between CALLY index quartiles and LVA risk across different clinical subgroups. **(A)** Age. **(B)** Sex. **(C)** BMI. **(D)** HBP. **(E)** Diabetes mellitus. **(F)** LVEF. LVA, left ventricular aneurysm; CALLY, C-reactive protein-albumin-lymphocyte; Q1–Q4, quartiles 1 through 4 of the CALLY index; BMI, body mass index; HBP, hypertension; DM, diabetes mellitus; LVEF, left ventricular ejection fraction.

Stratified analyses were performed to assess the consistency of the association between the CALLY index and LVA risk across clinical subgroups. As shown in Figure 5, LVA incidence was highest in CALLY Q1 and decreased gradually through Q4 in all subgroups. This trend was more obvious in patients with reduced left ventricular function: among patients with LVEF < 45%, LVA rates decreased from about 65% in Q1 to 22% in Q4, while among those with LVEF ≥ 45%, rates fell from about 10% to 2% (both *P* < 0.001). Similar stepwise decreases were observed across age, sex, BMI, HBP, and DM subgroups.

**Figure 6:**
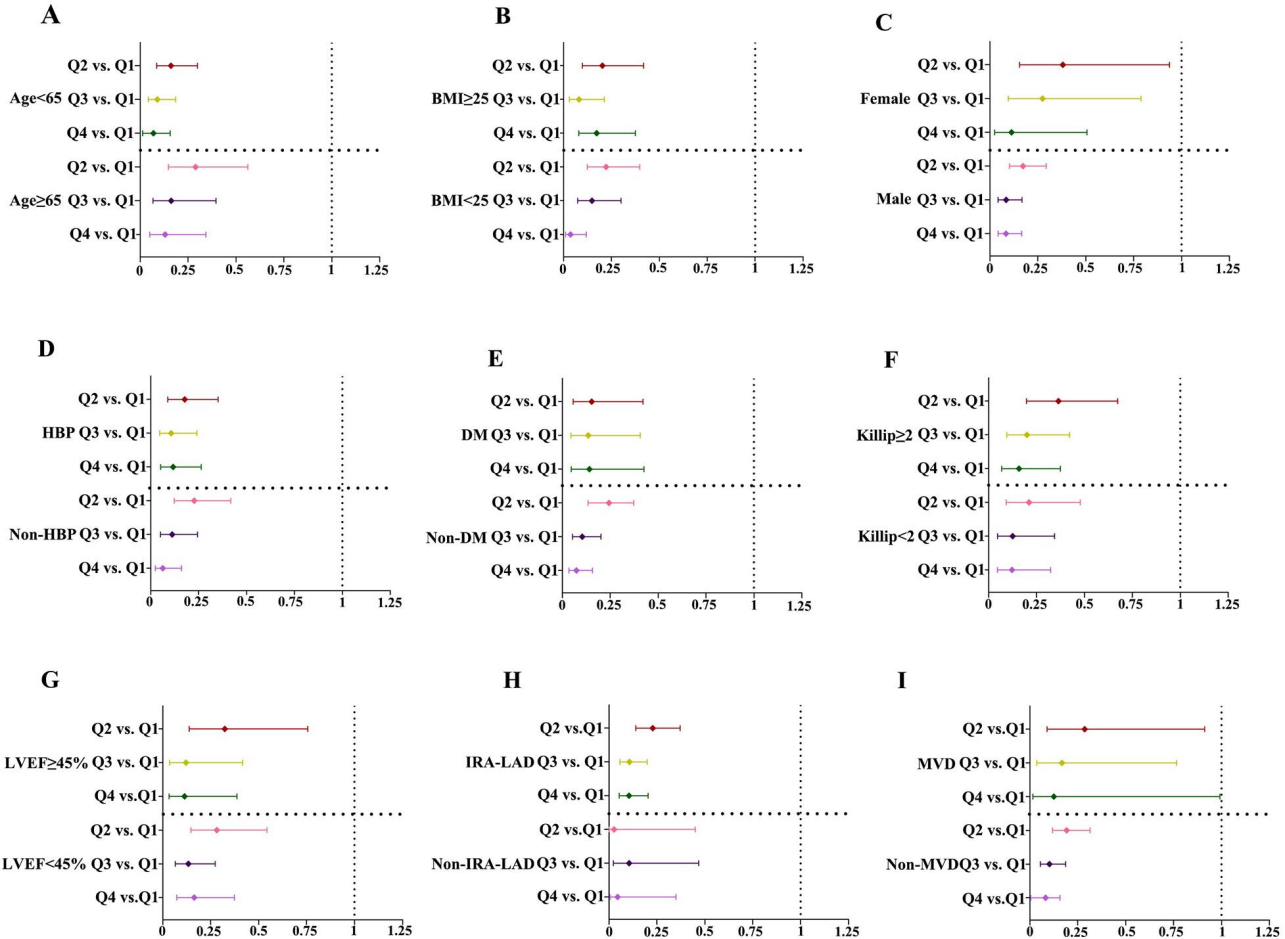
Subgroup analysis using forest plots to assess the association between CALLY quartiles and LVA risk. **(A)** Age. **(B)** BMI. **(C)** Sex. **(D)** HBP. **(E)** Diabetes mellitus. **(F)** Killip class (≥ 2 vs. < 2). **(G)** LVEF. **(H)** IRA-LAD. **(I)** MVD. Each panel presents the OR and 95% CI for LVA comparing Q2, Q3, and Q4 with Q1 (reference) within a specific subgroup. The vertical dotted line indicates the null value (OR=1.0). In all subgroups, higher CALLY quartiles were consistently associated with a lower risk of LVA, and formal interaction tests detected no significant effect modification by any stratifying variable. CALLY, C-reactive protein-albumin-lymphocyte index; LVA, left ventricular aneurysm; Q1–Q4, quartiles 1 through 4 of the CALLY index; OR, odds ratio; CI, confidence interval; BMI, body mass index; HBP, hypertension; DM, diabetes mellitus; LVEF, left ventricular ejection fraction; IRA-LAD, infarct-related artery as the left anterior descending artery; MVD, multivessel disease.

Forest plots were used to evaluate the stability of the association in each predefined subgroup (Figure 6). In Figure 6A, higher CALLY quartiles were associated with a lower risk of LVA in both patients aged <65 years and those ≥65 years. Figure 6B showed a similar pattern in both the BMI <25 kg/m^2^ and BMI ≥25 kg/m^2^ subgroups. Figure 6C demonstrated that the inverse association was present in both men and women, although the confidence intervals were somewhat wider in the female subgroup, reflecting the smaller event count.

Consistent findings were also observed in patients with and without HBP (Figure 6D), diabetes (Figure 6E), higher or lower Killip class (Figure 6F), reduced or preserved LVEF (Figure 6G), IRA-LAD or non–IRA-LAD (Figure 6H), and MVD or Non-MVD (Figure 6I).

Across all nine panels, the point estimates for Q2–Q4 remained below the null value of 1.0, and the majority of 95% CI excluded unity. Formal interaction tests detected no significant effect modification by any stratifying variable (all *P* for interaction > 0.05), suggesting that the association between CALLY index and LVA risk is consistent across a wide range of clinical characteristics in STEMI patients.

### Predictive performance and clinical utility

**Figure 7:**
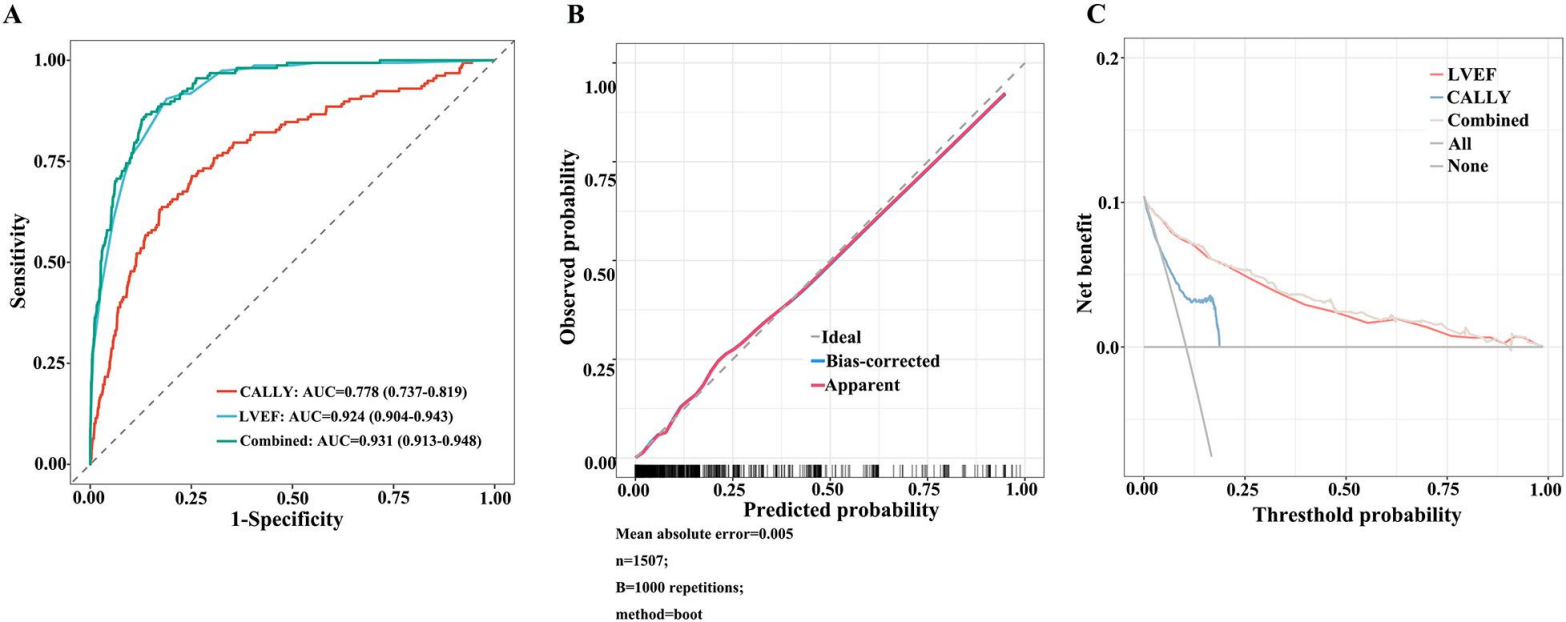
Predictive performance and clinical utility of the constructed prediction model. **(A)** ROC curves comparing the predictive ability of the standalone CALLY index, LVEF, and the final combined nomogram model for LVA risk. **(B)** Calibration curve of the combined nomogram. **(C)** DCA evaluating the net clinical benefit of different predictive strategies. ROC, receiver operating characteristic; AUC, area under the curve; CI, confidence interval; CALLY, C-reactive protein-albumin-lymphocyte index; LVEF, left ventricular ejection fraction; LVA, left ventricular aneurysm; DCA, decision curve analysis; boot, bootstrap resampling.

ROC analysis revealed that the CALLY index achieved an AUC of 0.778 (95% CI 0.737–0.819) for predicting LVA risk. For comparison, LVEF alone had an AUC of 0.924 (95% CI 0.904–0.943). The multivariable model combining the CALLY index with LVEF achieved an AUC of 0.931 (95% CI 0.913–0.948), suggesting improved discriminative performance compared with the CALLY index alone (Figure 7A).

Calibration of the multivariable model was evaluated using a calibration curve with 1,000 bootstrap resamples. Predicted probabilities corresponded well with observed probabilities, with a mean absolute error of 0.005 (Figure 7B).

DCA was conducted to assess the clinical utility of the models across different threshold probabilities. The combined model provided a positive net benefit across a wide range of clinically relevant thresholds and performed better than treat-all and treat-none strategies. LVEF also provided a positive net benefit, whereas the CALLY index showed a modest net benefit within a narrower threshold range (Figure 7C).

To directly compare CALLY with the previously reported MLR marker, we calculated MLR in the present cohort and performed ROC analysis. MLR alone showed a lower discriminatory ability for LVA prediction than CALLY alone (AUC: 0.659, 95% CI: 0.612–0.707 vs. 0.778, 95% CI: 0.737–0.819). When MLR was incorporated into an exploratory combined model, the AUC increased to 0.926 (95% CI: 0.907–0.944), which was close to but slightly lower than that of the final CALLY-based model (AUC: 0.931, 95% CI: 0.913–0.948) (Supplementary Figure 1).

### Nomogram development

**Figure 8:**
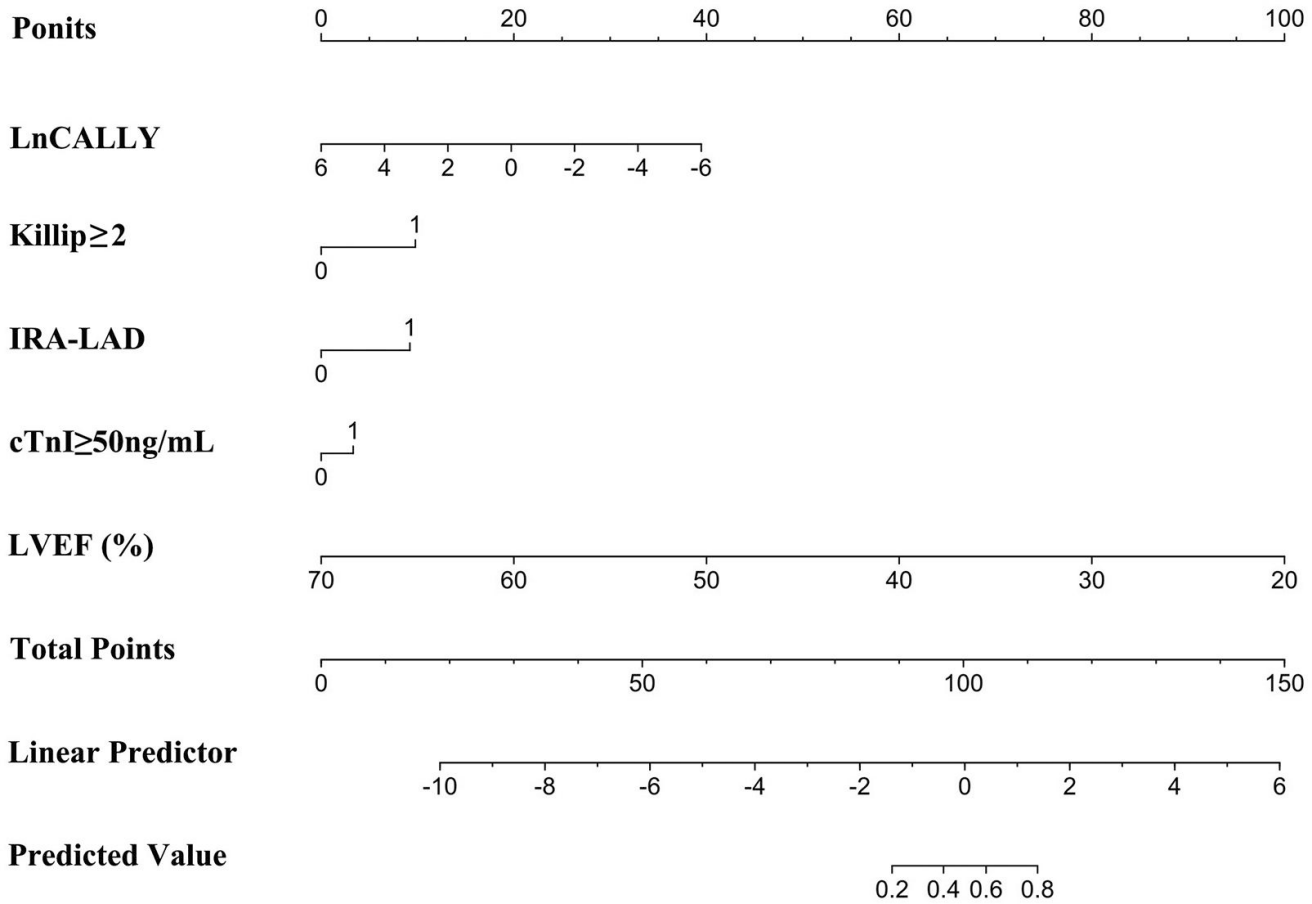
Nomogram for individualized prediction of LVA risk in STEMI patients undergoing pPCI. Ln, natural logarithm; CALLY, C-reactive protein-albumin-lymphocyte index; IRA-LAD, infarct-related artery – left anterior descending coronary artery; cTnI, cardiac troponin I; LVEF, left ventricular ejection fraction; STEMI, ST-segment elevation myocardial infarction; LVA, left ventricular aneurysm.

To provide a preliminary individualized risk estimate, a nomogram integrating ln(CALLY), Killip class ≥2, IRA-LAD, cTnI ≥50 ng/mL, and LVEF was constructed (Figure 8). Each predictor was assigned a point score on a 0-to-100 scale; the sum of points was linearly transformed into a predicted probability of LVA ranging from 0.2 to 0.8. For example, a patient with a low CALLY (ln-transformed value near −4), Killip class ≥ 2, IRA-LAD, elevated cTnI, and severely reduced LVEF would accumulate approximately 140–150 total points, corresponding to a predicted LVA probability exceeding 60%. Conversely, a patient with high CALLY, preserved LVEF, and none of the other risk factors would score near zero, with a predicted probability below 20%.

## Discussion

This study showed that a lower admission CALLY index was independently associated with a higher risk of LVA in STEMI patients treated with pPCI. The final multivariable model incorporating CALLY index and LVEF yielded an AUC of 0.931, with acceptable calibration and positive net benefit across a range of clinically relevant thresholds. These findings suggest that a composite immunonutritional biomarker may provide complementary information for preliminary identification of patients at higher risk of adverse ventricular remodeling.

It should be noted that LVEF alone showed excellent discrimination for LVA prediction in this cohort, and the increase in AUC from 0.924 to 0.931 after construction of the final multivariable model was limited. Therefore, the clinical value of the model should not be interpreted based on AUC improvement alone. Rather, its potential usefulness lies in its overall performance and complementary information. First, the calibration curve showed good agreement between predicted and observed probabilities, supporting its ability to provide individualized risk estimates. Second, DCA suggested a positive net benefit across clinically relevant threshold probabilities. Third, CALLY provides pathophysiological information related to systemic inflammation, nutritional reserve, and immune status, which differs from the structural and functional information reflected by LVEF. Thus, the CALLY-based multivariable model may complement, rather than replace, echocardiographic assessment and LVEF-based risk evaluation.

The biological basis for our findings is consistent with the key roles of inflammation, nutritional status, and immune function in post-infarction ventricular remodeling. CRP, an acute-phase protein, can promote myocardial injury by activating complement, recruiting leukocytes, and releasing matrix metalloproteinases, which may accelerate extracellular matrix breakdown and infarct expansion (22–24). Hypoalbuminemia reflects reduced hepatic synthetic function during the acute-phase response, as well as increased capillary permeability and oxidative stress, which can weaken the structural stability of the peri-infarct region (25). Lymphopenia, commonly observed after STEMI, mirrors an exhausted or sequestered adaptive immune compartment that impairs timely resolution of inflammation and tissue repair (26–28). Because albumin and lymphocyte count are placed in the numerator, whereas CRP is placed in the denominator, a lower CALLY index represents the combined presence of stronger systemic inflammation, reduced nutritional reserve, and impaired immune status. This biological pattern is consistent with a higher risk of adverse ventricular remodeling and LVA after STEMI.

The association between a lower CALLY index and LVA should also be interpreted in relation to infarct burden. A larger infarct may cause more extensive myocardial necrosis and a stronger systemic inflammatory response, accompanied by neurohumoral activation, metabolic stress, nutritional consumption, and immune dysregulation. These changes may collectively contribute to a lower CALLY index. Previous studies have shown that later or serial measurements of cardiac troponin are closely associated with infarct size and microvascular obstruction assessed by cardiac magnetic resonance imaging (29), while CRP levels after myocardial infarction are dynamic and have been associated with subsequent left ventricular dysfunction (30). Therefore, the lower admission CALLY index observed in patients who developed LVA may partly reflect the systemic inflammatory and immunonutritional response to a larger infarct burden, rather than acting as an isolated causal determinant of LVA. Although our model included several markers related to infarct severity, including cTnI, LVEF, IRA-LAD, and Killip class, residual confounding by unmeasured infarct size or microvascular obstruction cannot be fully excluded. Our RCS analysis further identified a nonlinear association, with LVA risk increasing notably when the CALLY index fell below approximately 1.0, suggesting a potential threshold for immunonutritional compromise.

Our results align with and extend prior evidence on the prognostic value of the CALLY index in cardiovascular disease. Ji et al. reported that a lower CALLY index was independently associated with short- and long-term major adverse cardiovascular events in 438 STEMI patients, with an AUC of 0.758 for short-term events (31). Similarly, Demir et al. found that a CALLY index ≤ 3.03 predicted in-hospital mortality after pPCI with 75.5% sensitivity and 78.2% specificity (32). Beyond the acute coronary syndrome setting, the CALLY index has demonstrated predictive utility in acute ischemic stroke (AUC 0.746 for hemorrhagic transformation) and in the general U.S. population for incident cardiovascular disease (33, 34). To our knowledge, the present study is the first to investigate the CALLY index as a predictor of LVA in STEMI patients. In our previous work, monocyte-to-lymphocyte ratio (MLR) was reported as a predictor of LVA after STEMI (35) In the present cohort, we further performed an exploratory head-to-head comparison. MLR alone showed a lower AUC than CALLY alone, whereas the MLR-based combined model achieved an AUC close to that of the CALLY-based final model. These findings suggest that CALLY may provide complementary information to MLR by integrating CRP, albumin, and lymphocyte count, thereby reflecting systemic inflammation, nutritional reserve, and immune status. However, whether CALLY is superior to MLR requires further validation in independent cohorts.

From a clinical perspective, the CALLY index is calculated from routine admission laboratory tests and can be obtained rapidly at no additional cost. The CALLY-based multivariable model may provide a preliminary individualized estimate of LVA risk and may complement echocardiographic assessment by reflecting systemic inflammatory, nutritional, and immune status. However, given the retrospective single-center design and the lack of external validation, this model should be regarded as exploratory and hypothesis-generating rather than a definitive clinical decision-making tool.

### Limitations

Several limitations should be acknowledged. First, this was a retrospective single-center study; although consecutive eligible STEMI patients treated with pPCI were screened, selection bias and limited generalizability cannot be fully excluded, especially because patients with missing essential data or loss to follow-up were excluded. Second, the number of LVA events was modest, which may affect the stability of the multivariable model and subgroup analyses. Third, the CALLY index was measured only at admission, whereas myocardial injury and inflammatory markers change dynamically after STEMI; therefore, the predictive value of serial CALLY measurements and residual confounding by unmeasured infarct size or microvascular obstruction could not be assessed. Fourth, LVA was primarily diagnosed by transthoracic echocardiography. Although a small number of patients underwent CMR imaging or left ventriculography during follow-up for clinical indications, these examinations were not systematically used. Therefore, mild, early, or delayed LVA may have been missed, and differentiation between true aneurysm and pseudoaneurysm relied mainly on echocardiographic assessment and clinical records. Finally, residual confounding by heart failure severity and post-STEMI medical therapy cannot be fully excluded. Although LVEF and Killip class were included in the final model, discharge medications such as diuretics, spironolactone, ACEI/ARB/ARNI, *β*-blockers, and statins were not incorporated because they were post-baseline treatment variables influenced by clinical severity and contraindications. Future prospective multicenter studies with longer standardized imaging follow-up are needed to validate these findings.

### Future directions

Future studies should validate the CALLY-based model in prospective multicenter cohorts with longer and standardized imaging follow-up. Serial CALLY measurements may help determine whether dynamic changes in inflammatory, nutritional, and immune status improve LVA risk assessment beyond a single admission value. In addition, integration with CMR imaging may further clarify the relationship between systemic immunonutritional status, infarct burden, and adverse ventricular remodeling.

## Conclusion

In conclusion, a lower admission CALLY index was independently associated with a higher risk of LVA in STEMI patients treated with pPCI. A multivariable model combining the CALLY index and LVEF demonstrated good discriminative performance (AUC = 0.931) and acceptable calibration, which was used to develop a clinically practical nomogram. These findings suggest that the CALLY index may provide complementary immunoinflammatory and nutritional information and may support preliminary risk stratification of LVA.

## Data Availability

The original contributions presented in the study are included in the article/Supplementary Material, further inquiries can be directed to the corresponding authors.
